# Primary osteosarcoma of the cranial vault

**DOI:** 10.1590/0100-3984.1914-2014

**Published:** 2017

**Authors:** Gabriel Lacerda Fernandes, Marcelo Ricardo Canuto Natal, Célio Lúcio Palha da Cruz, Rafael Lemos Nascif, Niedja Santos Gonçalves Tsuno, Marco Yukio Tsuno

**Affiliations:** 1 MD, Radiologist at Prodigy - Diagnóstico por Imagem, Brasília, DF, Brazil.; 2 MD, Radiologist, Head of the Radiology and Diagnostic Imaging Residency Program at the Hospital de Base do Distrito Federal (HBDF), Brasília, DF, Brazil.; 3 MD, Radiologist for Grupo Fleury - Hospital São Luiz, São Paulo, SP, Brazil.; 4 MD, Radiologist at Hospital Israelita Albert Einstein, Interventional Radiologist at the Hospital das Clínicas da Faculdade de Medicina da Universidade de São Paulo (HC-FMUSP)/Instituto do Câncer do Estado de São Paulo (Icesp), São Paulo, SP, Brazil.; 5 MD, Radiologist at Exame - Imagem e Laboratório, Brasília, DF, Brazil.

**Keywords:** Osteosarcoma, Neoplasms, Skull

## Abstract

Only 5-10% of osteosarcomas arise from the craniofacial bones. We report the case of a 14-year-old female patient who presented with headache and a mass that had been growing in the left frontoparietal region for six months. We describe the findings on conventional radiography, computed tomography, and magnetic resonance imaging.

## Abstract (pt)

Osteossarcomas que se originam dos ossos craniofaciais correspondem a apenas 5-10% dos casos. Neste artigo relatamos caso de uma paciente de 14 anos de idade com quadro de cefaleia e crescimento de massa tumoral na região frontoparietal esquerda com evolução de seis meses. São descritos os achados na radiografia simples, tomografia computadorizada e ressonância magnética.

## INTRODUCTION

Osteosarcoma accounts for approximately 20% of all primary bone malignancies. Of those, only 5-10% are located in the craniofacial bones accounts and most of those are found in the maxilla or mandible. Less than 1% are found in the cranial vault, and there have been only a few reported cases of osteosarcoma at the base of the skull, reflecting the low frequency of that location^(1-6)^. The osteoid and bone matrix of an osteosarcoma are composed of malignant connective tissue cells. Most osteosarcomas are of unknown cause and can therefore be designated idiopathic or primary^(1,2,4,6)^. Osteosarcomas related to known predisposing factors for malignancy, such as Paget’s disease, fibrous dysplasia, and external ionizing radiation, are referred to as secondary osteosarcomas^(1-7)^. The most common type of osteosarcoma is conventional osteosarcoma, the incidence of which is highest in patients in the second decade of life and the prevalence of which is slightly higher among males than among females^(1,2)^. In general, patients present with bone pain, occasionally accompanied by a mass or by soft tissue edema^(1,6)^. The distinct radiological aspects of conventional osteosarcoma are bone marrow lesions, cortical bone destruction, an aggressive periosteal reaction, a soft tissue mass, and a tumor matrix in the destructive lesion, as well as within the soft tissue mass. Although the tumors can present as purely sclerotic or purely osteolytic, most are a combination of the two. The borders are generally indistinct, with a broad zone of transition. The bone destruction is infiltrative, with a "moth-eaten" appearance, and only rarely geographic. The most common forms of periosteal reaction seen in osteosarcomas are the spiculated (sunburst) type and Codman’s triangle, the laminated (onion-skin) type being less common^(1,2,5-7)^.

## CASE REPORT

**Figure 1:**
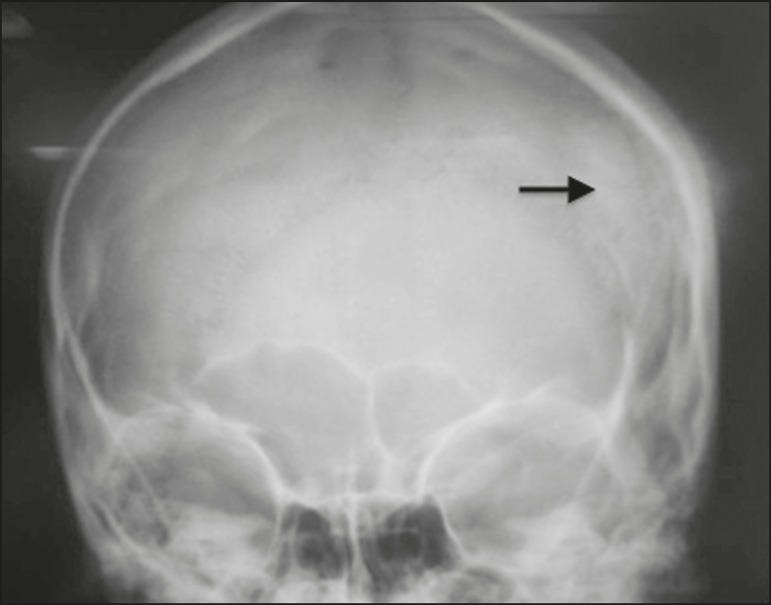
Anteroposterior X-ray of the skull, showing a heterogeneous high-intensity lesion in the left parietal region accompanied by an aggressive spiculated (sunburst) type periosteal reaction.

**Figure 2:**
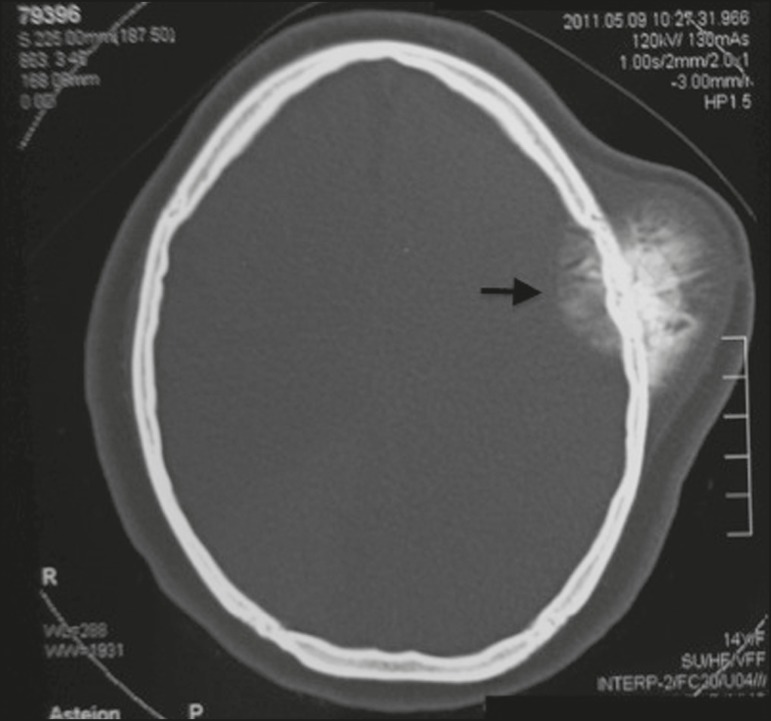
Computed tomography of the skull, showing a hyperintense intracranial/extracranial mass, with its epicenter in the cranial vault.

**Figure 3:**
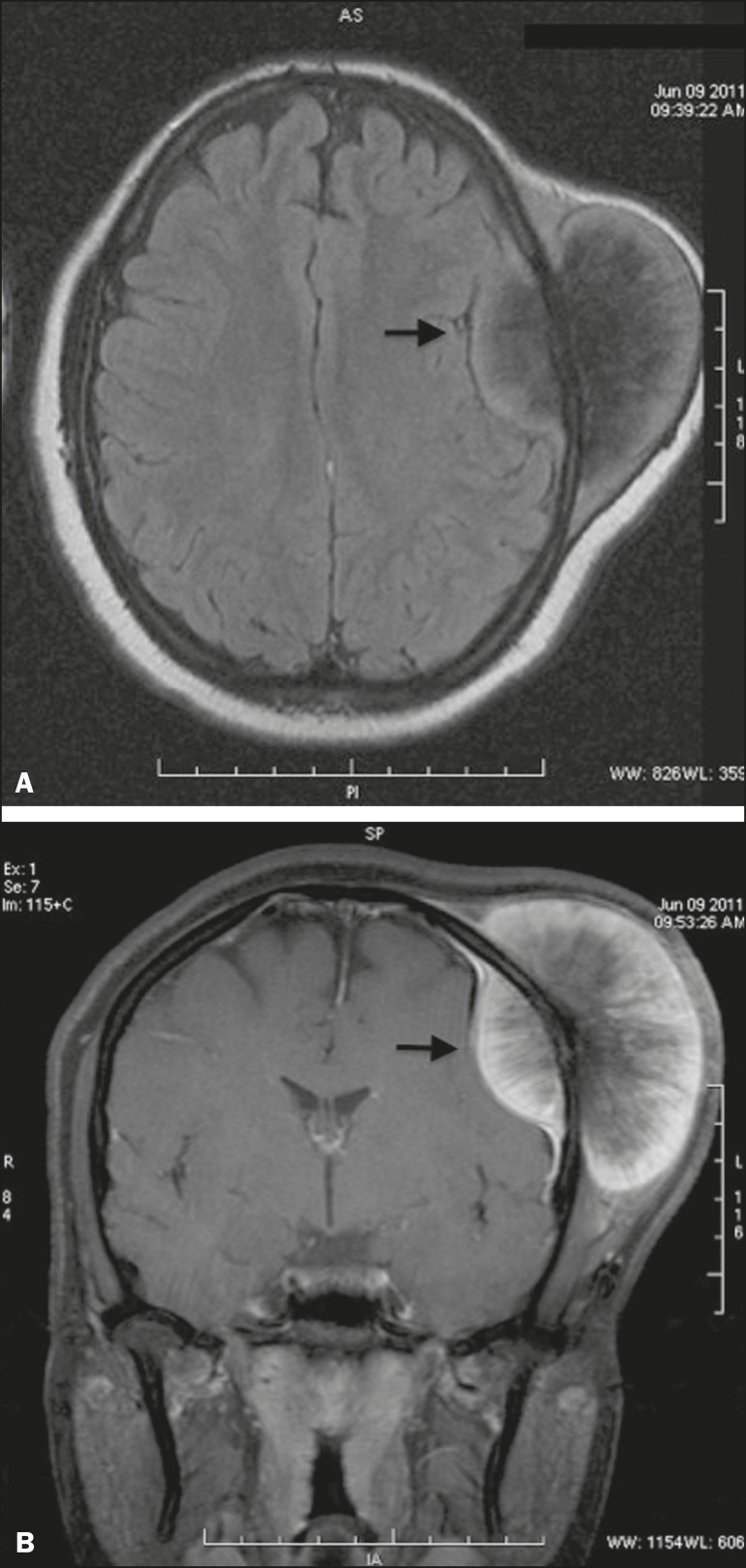
Contrast-enhanced magnetic resonance imaging of the skull-a axial T1-weighted sequence (**A**) and a fat-saturated coronal T1-weighted sequence (**B**)-showing a lesion with intracranial and extracranial components, its epicenter being in the cranial vault, and heterogeneous impregnation by the contrast medium, predominantly at its periphery.

A 14-year-old female patient presented with a headache and a mass that had been growing in the cranial vault for six months. The mass was hardened and was approximately 10 cm in diameter. An X-ray of the skull showed a discretely sclerotic lesion in the left parietal region, accompanied by an aggressive spiculated periosteal reaction (Figure 1). A computed tomography scan revealed a hyperintense mass in the left frontoparietal region, with intracranial and extracranial involvement (Figure 2). A contrast-enhanced magnetic resonance imaging scan showed an expansile lesion with intracranial and extracranial components, its epicenter being in the cranial vault. The lesion presented heterogeneous impregnation by the contrast medium, predominantly at its periphery (Figure 3). Evaluation of a biopsy sample identified malignant bone neoplasia that was classified as grade 3 (high grade) osteoblastic osteosarcoma. Therefore, the lesion was excised, with tumor-free margins, and part of the adjacent dura mater was resected because of suspicion of neoplastic involvement. The patient presented clinical improvement after the tumor resection and was discharged to outpatient follow-up.

## DISCUSSION

Here, we report a case of primary osteosarcoma of the skull, an extremely rare neoplasm at that location. Approximately 100 cases have been described in the literature^(2,4,5)^. The prognosis is worse than is that of osteosarcoma involving the bones of the appendicular skeleton, osteosarcoma of the skull presenting a lower response to aggressive multimodal therapy, with a five-year survival rate below 10%^(1-6)^.

In a clinical context in which the patient presents bone pain associated with a soft tissue mass, with suspected aggressive lesion, the investigation should begin with conventional radiology, because it can demonstrate important aspects of osteosarcoma, such as a periosteal reaction and cortical bone destruction. Computed tomography should be performed in order to characterize the dissemination of the tumor into the medullary cavity, as well as to provide images of the calcified neoplastic component, as well as of the involvement of the soft tissue and cortical bone, which are fundamental to the surgical planning. Magnetic resonance imaging has become an effective modality for evaluating such tumors, particularly for mapping the intraosseous/intracranial spread of the tumor, involvement of the soft tissues, and involvement of the neurovascular bundle. On T1-weighted images, the solid, non-mineralized portions of osteosarcoma generally appear as areas of low-to-medium signal intensity. On T2-weighted images, the tumor shows high signal intensity^(1,6)^. The typical pattern of contrast uptake by the lesion is one of intense heterogeneous impregnation.

The imaging-based differential diagnoses of osteosarcoma include hemangioma, which is characterized by multilocular lytic foci or coarse vertical striations; giant cell tumor, which is typically well-circumscribed but can cause thinning of the cortical bone and usually manifests as a lytic lesion; atypical meningioma, in which there is cortical bone destruction and extradural involvement; and metastases from cancer of the thyroid or digestive tract, which can present as expansile lesions accompanied by bone destruction and invading the soft tissues. Despite their similarity to osteosarcoma, all of those diseases are more commonly found in patients who are considerably older^(3,5,7,8)^.

Because of the rarity of the osteosarcoma of the skull, it is difficult to make the definitive diagnosis on the basis of imaging data. However, the knowledge that it is a destructive lesion associated with a soft tissue mass and an aggressive periosteal reaction can facilitate the diagnosis^(2)^. The treatment involves complete surgical resection of the lesion, with tumor-free margins, as well as chemotherapy and radiotherapy^(1-7)^.
